# Supplementary material for: Three-dimensional wide-field fluorescence microscopy for transcranial mapping of cortical microcirculation
Source: Nat Commun. 2022 Dec 28;13:7969. doi: 10.1038/s41467-022-35733-0 (PMC9797555; doi:10.1038/s41467-022-35733-0)
Supplement: Supplementary file 1 — Supplementary Information [file 41467_2022_35733_MOESM1_ESM.pdf]

Supplementary Information for

Three-dimensional wide-field fluorescence microscopy for transcranial mapping of cortical microcirculation

Quanyu Zhou,[1,2,†] Zhenyue Chen,[1,2,†] Yu-Hang Liu,[1,2] Mohamad El Amki,[3,4] Chaim Glück,[1,4] Jeanne Droux,[3,4] Michael Reiss,[1,2] Bruno Weber,[1,4] Susanne Wegener,[3,4] and Daniel Razansky[1,2,4,*]

[1]Institute of Pharmacology and Toxicology, Faculty of Medicine, University of Zurich, Switzerland

[2]Institute for Biomedical Engineering, Department of Information Technology and Electrical Engineering, ETH Zurich, Switzerland

[3]Department of Neurology, University Hospital and University of Zurich, Switzerland

[4]Zurich Neuroscience Center, Switzerland

*Correspondence
Daniel Razansky, Institute for Biomedical Engineering, Wolfgang-Pauli-Str. 27, 8093 Zurich, Switzerland
Email: daniel.razansky@uzh.ch

[†]These authors contributed equally

Supplementary Note 1: Evaluation of scattering impact on calibration curves in different scattering media

To study the impact of scattering on the calibration curve, a glass microscope slide with uniformly distributed cy5.5 dye was submerged in the Intralipid solution with concentrations of 0% (deionized water, non-scattering case), 0.5%, 1.0%, 1.5%, and 2.0%. These concentrations of Intralipid were chosen based on the fact that 1.5% Intralipid was commonly used to mimic the optical properties of biological tissue given a similar reduced scattering coefficient[1,2]. To build the calibration curve in scattering media, the fluorescence slide was shifted along z axis with a motorized stage starting from the zero-point relative to the microscope objective. The space between the zero-point and fluorescence slide was filled with the Intralipid solution. To create a flat Intralipid layer with a certain thickness, cover slips (85-115 µm thickness, CG00C2, Thorlabs, USA) were positioned and cascaded on both sides of the slide. The in-between cavity was filled with Intralipid solution with the given concentration and covered with an additional cover slip on the top. By cascading one additional cover slip on both sides, the thickness of Intralipid layer was increased by ~105 µm (mean value of randomly picked 20 cover slips), eventually resulting in an adjustable scattering distance from 0 to 525 µm with a step of 105 µm. For each Intralipid concentration, an image stack was collected to build the look-up table between the astigmatic point spread function (PSF) and the depth using four parameters including long-axis length w_x, short-axis length w_y, difference between both axes $w_x - w_y$, and ellipticity $\frac{w_x-w_y}{w_x+w_y}$ as defined in this work (supplementary Fig. 3a). With the increase of depth, the excitation power was increased following the exponential equation $I = I_0/\exp(-2\mu_t \mathrm{d})$ to compensate the fluorescence attenuation coming from the absorption and scattering of Intralipid where I_0 is the illumination power in the non-scattering case; μ_t is the total attenuation coefficient of Intralipid solution with given concentration at 660 nm wavelength; d is the scattering distance. The coefficient of 2 here is to take both the excitation and emission light path attenuation into consideration assuming the stokes shift between the excitation and emission wavelength of fluorophores is negligible. Although the

illumination power was compensated, the signal-to-noise ratio (SNR) still decreased with the increase of Intralipid concentration due to the strong background consisting of the scattered photons (supplementary Fig. 3b). To evaluate the robustness of the calibration curve in different scattering cases, coefficient of determination (R^2) was calculated between calibration curve in scattering cases and non-scattering. The averaged R^2 values from 156 illumination spots were shown in supplementary Fig. 3c. With the increase of Intralipid concentration, calibration curves based on all the selected parameters were featured with a decreased R^2. In 0.5% and 1.0% Intralipid cases, difference between both axes and ellipticity had higher R^2 values (close to 1) than long-axis length and short-axis length methods. In the 1.5% and 2.0% Intralipid cases, ellipticity-defined calibration curve showed significantly higher R^2 compared to another three methods, confirming the superiority of using ellipticity to build the calibration curve in scattering media.

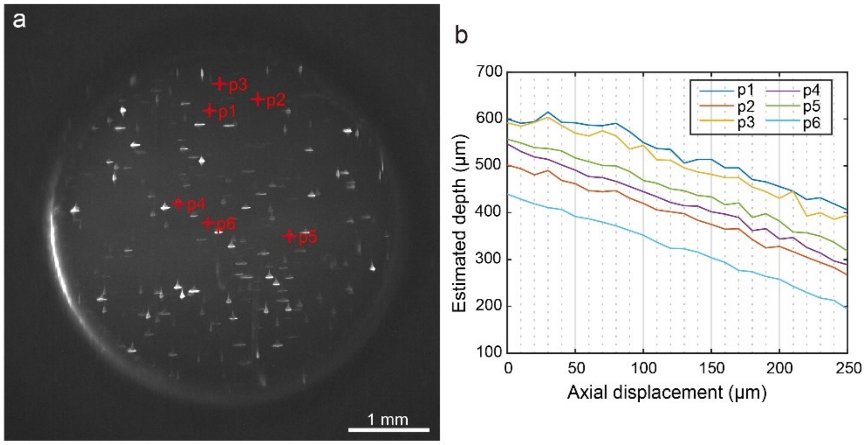

Supplementary Figure 1. 3D performance characterized by imaging sparsely distributed fluorescence beads in the UV-curing glue placed at different depth. (a) widefield image of fluorescence beads. (b) Estimated depth versus axial displacement of six beads selected from the center and edge in the field of view (FOV).

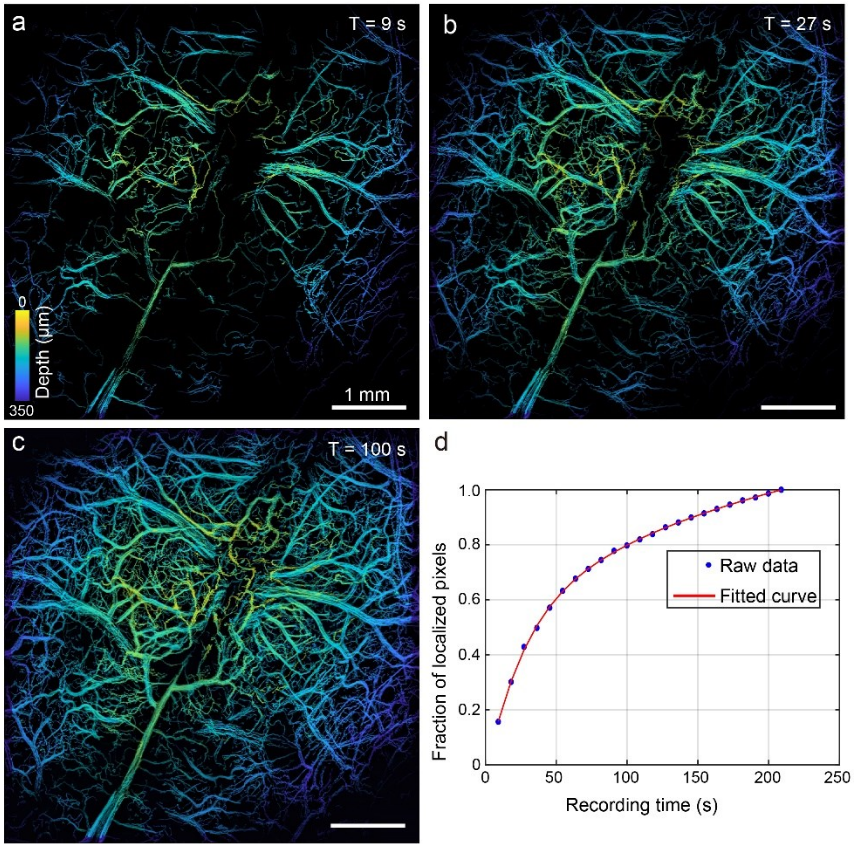

Supplementary Figure 2. Reconstructed SL image with different recording durations. (a-c) Corresponding SL image reconstructed from widefield image stack collected for 9, 27, and 100 s, respectively. (d) Fraction change of localized pixels versus recording time using the final localized image generated at 210 s as the reference. Polynomial fitting curve indicated that the fraction of localization pixels approached 80% at ~100 s.

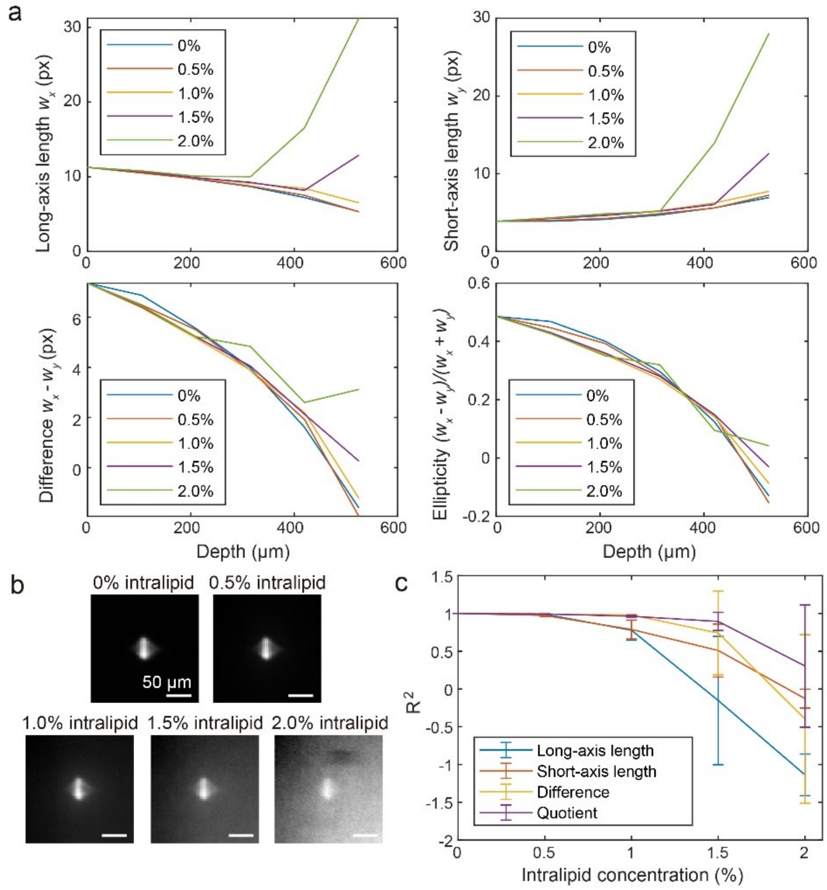

Supplementary Figure 3. Evaluation of scattering impact on calibration curves in different scattering media. (a) Calibration curves built with four different strategies (long-axis length, short-axis length, difference between both axes, and ellipticity) as a function of sample depth inside 0%, 0.5%, 1.0%, 1.5%, and 2.0% concentration (v/v) Intralipid solution, respectively. (b) Representative images of PSF at different Intralipid concentrations. (c) R^2 calculated between calibration curves in scattering cases and non-scattering case. Mean and standard deviation values were calculated from 156 illumination spots within the FOV.

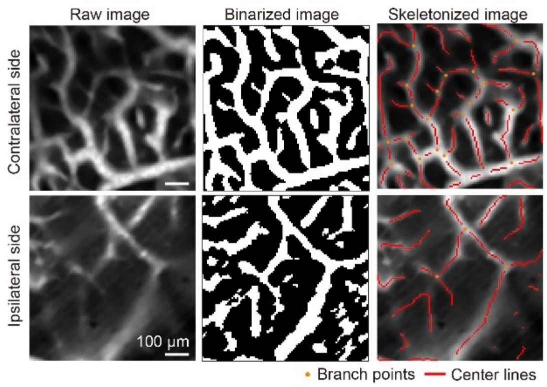

Supplementary Figure 4. Quantification of vessel parameters (i.e., fill fraction, number of vessel branches) using automatic vessel segmentation and analysis algorithm. The workflow could be simplified into three steps: (1) pre-processing to enhance the image contrast using image-guided and Frangi filter; (2) image binarization based on Otsu's algorithm; (3) vessel centerline computation through the morphological thinning algorithm followed by recognizing branch points based on the number of non-zero neighborhood pixels. The same algorithm and setting were used to quantify three region of interest (ROI) pairs selected from both the contralateral and ipsilateral side in the mouse brain post ischemia stroke.

References:

1 Lai, P., Xu, X. & Wang, L. V. Dependence of optical scattering from Intralipid in gelatin-gel based tissue-mimicking phantoms on mixing temperature and time. *Journal of biomedical optics* **19**, 035002 (2014).

2 Hong, G. *et al.* Through-skull fluorescence imaging of the brain in a new near-infrared window. *Nature photonics* **8**, 723-730 (2014).
