# Supplementary material for: Three-dimensional wide-field fluorescence microscopy for transcranial mapping of cortical microcirculation
Source: Nat Commun. 2022 Dec 28;13:7969. doi: 10.1038/s41467-022-35733-0 (PMC9797555; doi:10.1038/s41467-022-35733-0)
Supplement: Supplementary file 3 — Description of Additional Supplementary Files [file 41467_2022_35733_MOESM3_ESM.docx]

**Description of Additional Supplementary Files**

**Supplementary Movie 1:** 3D cortical microcirculation mapping in the mouse brain with sparse localization (SL) method.

**Supplementary Movie 2:** Time-lapse dye perfusion recording in the mouse brain post ischemia stroke captured with multifocal illumination (MI) method.
