# Supplementary material for: Three-dimensional wide-field fluorescence microscopy for transcranial mapping of cortical microcirculation
Source: Nat Commun. 2022 Dec 28;13:7969. doi: 10.1038/s41467-022-35733-0 (PMC9797555; doi:10.1038/s41467-022-35733-0)
Supplement: Supplementary file 6 — Reporting Summary [file 41467_2022_35733_MOESM6_ESM.pdf]

Corresponding author(s): Daniel Razansky

Last updated by author(s): Nov 27, 2022

Reporting Summary

Nature Portfolio wishes to improve the reproducibility of the work that we publish. This form provides structure for consistency and transparency in reporting. For further information on Nature Portfolio policies, see our Editorial Policies and the Editorial Policy Checklist.

Statistics

For all statistical analyses, confirm that the following items are present in the figure legend, table legend, main text, or Methods section.

n/a	Confirmed	
☐	☒	The exact sample size (*n*) for each experimental group/condition, given as a discrete number and unit of measurement
☒	☐	A statement on whether measurements were taken from distinct samples or whether the same sample was measured repeatedly
☐	☒	The statistical test(s) used AND whether they are one- or two-sided<br>*Only common tests should be described solely by name; describe more complex techniques in the Methods section.*
☒	☐	A description of all covariates tested
☒	☐	A description of any assumptions or corrections, such as tests of normality and adjustment for multiple comparisons
☐	☒	A full description of the statistical parameters including central tendency (e.g. means) or other basic estimates (e.g. regression coefficient) AND variation (e.g. standard deviation) or associated estimates of uncertainty (e.g. confidence intervals)
☐	☒	For null hypothesis testing, the test statistic (e.g. *F*, *t*, *r*) with confidence intervals, effect sizes, degrees of freedom and *P* value noted<br>*Give P values as exact values whenever suitable.*
☒	☐	For Bayesian analysis, information on the choice of priors and Markov chain Monte Carlo settings
☒	☐	For hierarchical and complex designs, identification of the appropriate level for tests and full reporting of outcomes
☐	☒	Estimates of effect sizes (e.g. Cohen's *d*, Pearson's *r*), indicating how they were calculated

Our web collection on statistics for biologists contains articles on many of the points above.

Software and code

Policy information about availability of computer code

Data collection	Camware64 (version 4.12, PCO AG, Germany) and MoorFLPI software (version 4.0, Moor Instrument, UK) were used for data collection.
Data analysis	Custom code used for image reconstruction is available for research purposes from the corresponding author upon request. Simpletracker (no version number available, https://github.com/tinevez/simpletracker, 2019) was used for image reconstruction. PostProGUI (version 1.0, https://github.com/razanskylab/PostProGUI, 2021) was used for automatic vessel analysis. MATLAB R2019b (MathWorks, MATLAB R2019b, USA) was used for data analysis.

For manuscripts utilizing custom algorithms or software that are central to the research but not yet described in published literature, software must be made available to editors and reviewers. We strongly encourage code deposition in a community repository (e.g. GitHub). See the Nature Portfolio guidelines for submitting code & software for further information.

Data

Policy information about availability of data

All manuscripts must include a data availability statement. This statement should provide the following information, where applicable:

- Accession codes, unique identifiers, or web links for publicly available datasets
- A description of any restrictions on data availability
- For clinical datasets or third party data, please ensure that the statement adheres to our policy

The main data supporting the finding of this study are available within the main text or supplementary information. Raw data for Figs. 1b-c, 2c, 2j, 4e, 4g and supplementary Figs. 1b, 2d, 3a, 3c are provided in the Source Data file. The raw datasets before image reconstruction are too large to be publicly shared, yet they

are available for research purposes from the corresponding author upon request.

Human research participants

Policy information about studies involving human research participants and Sex and Gender in Research.

Reporting on sex and gender	Not applicable
Population characteristics	Not applicable
Recruitment	Not applicable
Ethics oversight	Not applicable

Note that full information on the approval of the study protocol must also be provided in the manuscript.

Field-specific reporting

Please select the one below that is the best fit for your research. If you are not sure, read the appropriate sections before making your selection.

[x] Life sciences [ ] Behavioural & social sciences [ ] Ecological, evolutionary & environmental sciences

For a reference copy of the document with all sections, see nature.com/documents/nr-reporting-summary-flat.pdf

Life sciences study design

All studies must disclose on these points even when the disclosure is negative.

Sample size	No sample-size calculation was performed since the purpose of the experiments in Figure 2 and 3 is to characterize the imaging performance of proposed method and cross-validation with another modality (two-photon microscopy), which is expected to be similar on different animals. Thus, these experiments were performed with one mouse for each. The stroke experiment in Figure 4 was repeated with 3 mice with similar results. Representative results from one mouse was used in Figure 4. For statistical analysis, different vessels and region of interests (ROIs) were chosen independently in the same mouse for data analysis with sample size labeled in the figure legend.
Data exclusions	No data exclusion.
Replication	The animal experiments in Figure 2 and 3 is to characterize the imaging performance which was be performed with one mouse for each experiment. As the manuscript focused on the demonstrating the 3D performance of proposed microscopy technique rather than reaching biological conclusions, we did not extensively repeat the experiments. The stroke experiment in Figure 4 was repeated with 3 mice which showed consistent results.
Randomization	Randomization was not relevant to this study since the imaging performance was independent of animals.
Blinding	Blinding was not relevant to this study since the imaging performance was independent of animals.

Reporting for specific materials, systems and methods

We require information from authors about some types of materials, experimental systems and methods used in many studies. Here, indicate whether each material, system or method listed is relevant to your study. If you are not sure if a list item applies to your research, read the appropriate section before selecting a response.

Materials & experimental systems

n/a	Involved in the study	
[x]	[ ]	Antibodies
[x]	[ ]	Eukaryotic cell lines
[x]	[ ]	Palaeontology and archaeology
[ ]	[x]	Animals and other organisms
[x]	[ ]	Clinical data
[x]	[ ]	Dual use research of concern

Methods

n/a	Involved in the study	
[x]	[ ]	ChIP-seq
[x]	[ ]	Flow cytometry
[x]	[ ]	MRI-based neuroimaging

Animals and other research organisms

Policy information about studies involving animals; ARRIVE guidelines recommended for reporting animal research, and Sex and Gender in Research

Laboratory animals	In total, two female athymic nude-Fox1nu mice (8 weeks old and 12 weeks old, Envigo BMC B.V., Netherlands), two female C57BL/6J mice (7 weeks old and 10 weeks old, Envigo BMC B.V., Netherlands), and two male C57BL/6J mice (6 weeks old, Envigo BMS B.V., Netherlands) were involved in this study. Animals were housed in ventilated cages inside a temperature-controlled room under a 12 h dark/light cycle. The temperature was kept at 22 degree Celsius and relative humidity was kept at 50%. Pelleted food (3437PXL15, Cargill) and water were provided.
Wild animals	No wild animals were used in the study.
Reporting on sex	Both male and female mice were used in the imaging experiment.
Field-collected samples	No field-collected samples were used in the study.
Ethics oversight	All animal experiments were performed in accordance with the Swiss Federal Act on Animal Protection and approved by the Cantonal Veterinary Office Zurich (permit ZH161/2018, ZH165/2019).

Note that full information on the approval of the study protocol must also be provided in the manuscript.
